# PReS-FINAL-2155: Genetic variability of methotrexate transporters in patients with juvenile idiopathic arthritis

**DOI:** 10.1186/1546-0096-11-S2-P167

**Published:** 2013-12-05

**Authors:** M Zajc Avramovič, N Toplak, M Debeljak, M Accetto, L Lusa, V Dolžan, T Avcin

**Affiliations:** 1 Paediatric Clinic of University Clinical Centre Ljubljana, Ljubljana, Slovenia; 2 Institute for Biostatistics and Medical Informatics, University of Ljubljana, Faculty of Medicine, Ljubljana, Slovenia; 3 Institute of Biochemistry, Faculty of Medicine, University of Ljubljana, Ljubljana, Slovenia

## Introduction

Factors that would predict treatment outcome for methotrexate (MTX) would be of great value to clinicians. Recent pharmacogenetic studies have reported associations between single nucleotide polymorphisms (SNP) in MTX transporters and treatment outcome in childhood acute lymphoblastic leukemia and in rheumatoid arthritis.

## Objectives

To investigate the influence of SNP in the genes for MTX uptake and efflux transporters on toxicity and response to therapy in JIA.

## Methods

The data of 77 consecutive patients with JIA treated with MTX at the University Children's Hospital Ljubljana from June 2011 to February 2013 have been retrospectively reviewed. The disease activity was measured by JADAS 71 score 3 and 6 months after the beginning of treatment with MTX and at the last follow up visit. All adverse events were noted separately for different organ systems. Genotyping of single nucleotide polymorphisms (SNP) in the genes of MTX transporters was performed using real time PCR methods. The analysed SNPs were: ABCB1 3435C>T (rs1045642), ABCC2 24C>T (rs717620), ABCC2 1019A>G (rs2804402), ABCC2 1249G>A (rs2273697), ABCG2 34G>A (rs2231137), ABCG2 421C>A (rs 2231142), SLCO1B1 174Ala>Val (rs4149056), SLCO1B1 388 A>G (rs2306283), SLCO1B1 int13 T>C (rs11045879) and SLC19A1 (RFC1) 80G>A (rs1051266). Chi-square test and logistic regression were used for the statistical analysis.

## Results

The study group included 54 girls (70%) and 23 boys (30%) with JIA with mean disease duration 63 months. Nine (12%) patients had systemic arthritis, 23 (30%) patients had polyarthritis (4 out of these were RF positive), 15 (19%) patients had persistent oligoarthritis, 14 (18%) extended oligoarthritis, 10 (13%) patients had juvenile psoriatic arthritis and one (1%) patient suffered from enthesitis related arthritis. Five (6%) patients were treated with MTX because of chronic idiopathic uveitis. Mean follow up time was 80 months. Thirteen out of 77 (17%) patients were in remission without therapy at the last follow up visit. In total 37 out of 77 patients (48%) had to be switched to biologic therapy due to treatment inefficacy or severe adverse events. Adverse events developed in 47 patients (61%), 11 patients (14%) had severe adverse events and 9 patients (12%) discontinued MTX treatment because of adverse events. SLCO1B1 174Ala>Val (p = 0.059), ABCB1 3435C>T (p = 0.063 OR:3.065, 95%CI:0.908-10.338) and ABCC2 1019A>G (p = 0.113, OR:3.592, 95% CI: 0.739-17.461) showed a trend for association with gastrointestinal adverse events. ABCG2 34G>A (p = 0.054) and SLC19A1 80G>A (p = 0.078) were marginally associated with dermatological adverse events, while ABCG2 34G>A showed association with infections (p = 0.049 OR:5.158, 95%CI: 0.871-30.528).

## Conclusion

We reported for the first time the influence of SLCO1B1 on MTX treatment toxicity in JIA. SNPs in MTX transporters' genes may be a useful tool to predict toxicity in patients with JIA treated with MTX, but replications of our study in larger groups of patients are needed.

## Disclosure of interest

None declared.

